# Developmental basis of natural tooth shape variation in cichlid fishes

**DOI:** 10.1007/s00114-025-01964-6

**Published:** 2025-01-27

**Authors:** Ryan F. Bloomquist

**Affiliations:** 1 Institute of Bioengineering and Biosciences, School of Biological Sciences, Georgia Institute of Technology, Atlanta, GA USA; 2 School of Medicine, University of South Carolina, 6311 Garners Ferry Rd, Columbia, SC 29209 USA

**Keywords:** Enamel knots, Tooth shape, Cichlids

## Abstract

While most dentate non-mammalian vertebrates possess simple conical teeth, some demonstrate complex tooth shapes. Lake Malawi cichlid fishes are an extreme example of this, exhibiting a myriad of tooth shapes driven by an ecologically derived rapid evolution of closely related but distinct species. Tooth shape in mammals is generally considered to be established by signaling centers called primary and secondary enamel knots, which are not believed to be present in non-mammalian vertebrates. In this study, signaling centers of gene expression with epithelial folding with similar molecular patterns to that of mammalian enamel knots are identified, and differences of asymmetric gene expression are identified between fish that possess species specific polymorphisms of either bicuspid or tricuspid teeth. Gene expression is then manipulated indirectly using a small molecule inhibitor of the Notch pathway, resulting in phenotypical aberrations of tooth shape and patterning, including a mimic of a tricuspid tooth in a fish with a naturally occurring bicuspid dentition. This study provides insight into the evolutionary origins of tooth shape and advances our knowledge of the molecular determinants of dental morphology with translational utility in regenerative dentistry.

**Supplementary Information:**

The online version contains supplementary material available at 10.1007/s00114-025-01964-6.

## Introduction

Teeth are some of the most morphologically diverse hard tissues across vertebrates. These structures vary greatly in size, shape, and pattern across species, yet are remarkably patterned precisely and predictably within species. Diversity in tooth shape in mammals is evolutionarily driven by the complexity of their diets and the ecological niches they occupy (Jernvall 1997; Ungar 2010). In contrast, the majority of non-mammalian vertebrates including reptiles and teleosts have relatively simple conical teeth (Herzberg & Massler 1940; Huysseune & Sire 1998; Ungar 2010) or rudimentary odontodes (Fraser et al. 2010) that are regularly turned over through tooth replacement (Tucker & Fraser 2014). This is not always the case however, as some non-mammalian species such as sharks, pufferfish, and cichlid fish have evolved specialized dentitions that meet ecological adaptations (Frazzetta 1988; Streelman et al. 2003; Fraser et al. 2012, 2013). While dental phenotypes vary widely across different species (Van Valen 1965), tooth shapes are highly constrained within a given species, suggesting tight genetic and developmental regulation of tooth formation (Evans & Sanson 2003). Only in recent years have we begun to understand the complex genetic and tissue interactions that govern the form of teeth. As medicine moves towards the capability of regenerating teeth, there is a greater need for understanding the intricacies of how teeth are formed and what molecular mechanisms influence tooth shape.

In many mammals, there is shape diversity within a row of teeth, known as heterodonty. Fields of gene expression have been identified that create a spatial hierarchy, wherein different tooth shapes are determined (Tucker & Sharpe 2004). For example, in mice, regenerating asymmetric and unicuspid incisors develop in a bone morphogenetic protein (BMP)–rich epithelial field of the anterior oral cavity while non-regenerating molars with complex forms develop in the fibroblast growth factor (FGF)–rich epithelial region of the posterior oral cavity (Tucker et al. 1998; Tucker & Sharpe 2004). Intriguingly, when BMPs are downregulated through implantation of Noggin soaked beads, molars grow in the anterior presumptive incisors field, suggesting early molecular signals at initiation of tooth development are enough to pattern the downstream signaling cascades for late dental morphogenesis (Tucker et al. 1998). These are not the only time points or factors of odontogenesis that can influence shape, and a series of complex interactions help a tooth take its final form (Tucker et al. 1998). Mathematical models reveal that any number of modifications in signaling factors integrated with cellular parameters have a strong influence on tissue mechanics and help give rise to the complexity of tooth types in seal dentitions and likely all animals with complex dentitions (Salazar-Ciudad & Jernvall 2010).

As a tooth is formed, it goes through stages of increasing differentiation and hard tissue secretion. Initiating as a thickening of epithelium, the tooth bud begins to signal underlying neural crest-derived mesenchyme to condense. In mammals, these two layers will reciprocally influence each other to fold as they progress through the cap, then the bell, and finally hard tissue secretion stages (Butler 1956; Peterkova et al. 2000). The tooth bud then enters the cap stage, where the epithelium differentiates to create layers known as inner enamel epithelium, stellate reticulum, stratum intermedium, and outer enamel epithelium. It is at this point that the number of cusps and the overall shape of the tooth begins to materialize. In mammals such as the mouse, signaling centers in the dental epithelium known as enamel knots develop at the cap stage with distinctive histology and molecular signatures (Butler 1956; Jernvall et al. 1994; Vaahtokari et al. 1996a). As a developing tooth progresses from the cap to bell to secretion stages, these transient structures are further subdivided into primary, secondary, and even tertiary enamel knots in order of their appearance and disappearance (Luukko et al. 2003). Primary knots have been observed in mouse incisors with simple unicuspid shape and, even while the molecular signature persists into the adult life of the rodent incisors (Nakatomi et al. 2015), incisors lack secondary and tertiary knots responsible for additional cusp formation. As opposed to the epithelium surrounding knots that is rapidly proliferating during tooth development, knots are non-proliferative and secrete distinct ligands such as *Fgf4* in secondary knots (Jernvall et al. 1994), and further serve as centers of apoptosis (Vaahtokari et al. 1996b). This complex concert of delineation from the native uniform epithelium gives rise to the spatial zones that will create cusps, fossa, and grooves of teeth. It is important, however, to point out that these complex patterns of knot-driven signaling centers have only been described in mammals, such as mouse (Jernvall et al. 1994) and ferret (Jussila et al. 2014). In fishes and reptiles, there has been a general consensus that enamel knots do not exist.(Handrigan & Richman 2011; Richman & Handrigan 2011; Debiais-Thibaud et al. 2015b, Landova Sulcova et al. 2020) However, signaling centers that are very similar to mammalian knots have been described, including nodes of *bmp* and *shh* expression with slow cellular proliferation in the tips of developing catshark teeth (Debiais-Thibaud et al. 2015b). In squamates, complex tooth shape diversity has been described including biscuspid and tricuspid teeth across species but a genetic basis for this diversity is not yet clear (Zahradnicek et al. 2014). Studies have highlighted analogous signaling centers and zones of non-proliferation in the inner enamel epithelium of bearded dragons (Handrigan & Richman 2010), pythons (Buchtová et al. 2008), and geckos (Handrigan et al. 2010), but these centers were not considered to be enamel knots (Richman & Handrigan 2011). Another recent study provides clear evidence of signaling centers in shark teeth and while not completely identical to mammalian enamel knots, the authors suggest that these signaling centers for shape determination may predate even teeth themselves (Thiery et al. 2022). It is clear that similar pathways acting in the formation of mouse tooth shape are evident in non-mammalian vertebrates with complex teeth. For instance, modulation by either downregulating the Bmp pathway or upregulating the Fgf pathways in zebrafish result in supernumerary teeth and additional cusp formation (Jackman et al. 2013). These experiments correlate nicely with mouse experiments wherein molars develop in FGF rich fields and incisors develop in BMP rich fields (Tucker et al. 1998). There has been little evidence of apoptosis in these signaling centers, an important enamel knot identifier in mice (Jernvall et al. 1998), which may be due to the technical difficulties of doing these studies in novel systems. For instance, in cichlids, paraffin sections of the jaw are difficult because of the high spatial density of teeth and bone, and many experiments are done with vibratome sectioning which limits the types of post-histology staining available (Fraser et al. 2013). Some studies suggest that altered apoptosis does not influence crown morphology, and perhaps, this pathway is playing a different role in mouse molar development (Coin et al. 2000). If ecologically driven dental morphological complexity exists in non-mammalian vertebrates, it is possible that they possess unique systems of producing dental morphology when compared to that of mammals, but it is more likely that these have not yet observed the mechanisms of tooth formation in the same level of detail as that of the mouse. Moreover, with the number of reports of knot-like signaling centers in non-mammalian vertebrates mounting, the argument that shape determining knots do not exist in these animals is growing thinner by the year.

There are several pathways that have been indicated in shape determination and enamel knot signaling of mammals. The Bmp family of growth factors, including *bmp2*, are known knot markers and are expressed in either primary, secondary, or tertiary knots (Meguro et al. 2019). BMPs are regulated by factors such as the BMP family inhibitor *Follistatin (Fst*), which is expressed in the knot of mice, and in transgenic *Fst* nulls demonstrate severe dental shape abnormalities (Wang et al. 2004). SHH is another well-known ligand that patterns organs and is expressed in both mouse and ferret enamel knots (Jussila et al. 2014). The FGF pathway is also a key player in tooth shape specification, and a host of studies demonstrate that FGF ligands function in a robust and redundant manner to specify dental morphogenesis through pathway interactions and apoptosis regulation (Jernvall et al. 1994; Vaahtokari et al. 1996b; Harada et al. 1999; Mustonen et al. 2002). The WNT signal transduction is one of the most well studied enamel knot signaling pathways, with *Wnt5a* being expressed in mammals including mice (Lin et al. 2011) to mini-pigs (Wu et al. 2017), and with *Wnt5a* mutant mice expressing upregulated *Shh* and a resulting abnormal tooth morphology (Lin et al. 2011). These genes interact and are co-regulated and interdependent, with single genes such as the BMP inhibitor *Ectodin* being positively regulated by BMPs, while negatively regulated by FGFs and SHH in reciprocal feedback loops of the enamel knot to help define spatial organization of cell layers in tooth explants (Laurikkala et al. 2003). Taken together, the molecular genetic determinants of tooth shape are complex and work in a concert.

Lake Malawi cichlid fish afford a powerful model for studying natural polymorphism in tooth shape. Lake Malawi cichlid fish are one of the greatest examples of adaptive radiation in nature (Brawand et al. 2014b), making them an ideal model for studying the genetic basis of morphological polymorphisms (Kocher 2004). Their jaws and dentitions help drive interspecific diversity, with closely related species exhibiting a kaleidoscopic of jaw morphologies and related unicuspid, bicuspid, and tricuspid tooth shapes (Fryer & Iles 1972; Vandervennet et al. 2006; Fraser et al. 2008, 2013). Teleosts and other non-mammalian vertebrates such as cichlids do not possess enamel-proper, but rather a homologous hydroxyapatite hard dental tissue known as enameloid. Our group has previously observed Malawi cichlid dental development at histological and signaling levels and have noted enameloid-knot like centers at the tips of teeth (Fraser et al. 2013), but we have not characterized them or studied differences between closely related cichlid species that represent an array of different tooth shapes. Additionally QTL has been used to identify genetic linkage associations for differences in tooth shape between cichlid species that have bicuspid teeth *Metriaclima zebra* (MZ) and those that only possess tricuspid teeth *Labeotropheus fuelleborni* (LF) (Streelman & Albertson 2006). These closely related species are under different trophic constraints and have nearly identical genomes (Loh et al. 2008), but obligate distinct dentitions. Two regions of the cichlid genome segregate to tooth shape, located in a 3.7-cM interval on cichlid linkage group 5, and several candidate genes for tooth shape determination are located here, including Basic Helix-Loop-Helix Family Member E40 (*bhlhee40*) examined in this study (Streelman & Albertson 2006; Brawand et al. 2014b). In this present analysis, a molecular analysis through in situ hybridization (ISH) was performed to uncover differences in signaling patterns between species with bicuspid or tricuspid teeth and the number of signaling centers present. These genes included members of the bone morphogenetic protein (Bmp), hedgehog (Shh), fibroblast growth factor (Fgf), wingless (Wnt), and Notch gene families. The Notch pathway was then chemically antagonized, which has been shown to help determine tooth shape (Mustonen et al. 2002; Mitsiadis et al. 2010). These data provide insight to the evolution of tooth shape and help decipher the primal modulators of dental architecture in cichlids.

## Materials and methods

### Cichlid husbandry

Adult Malawi cichlids were housed in re-circulating aquarium systems at 28 °C (GIT) for embryo production. Species of Lake Malawi cichlids include *Labeotropheus fuelleborni* (LF), *Metriaclima zebra* (MZ), and *Petrotilapia chitimba* (PC) and were selected based on embryo availability with a preference toward MZ, owing to their genome assemblage (55) and partial albinism morph which permitted better imaging of histological stain. LF were selected for study as an obligate homodont tricuspid dentition, while MZ selected for their outer row obligate bicuspid dentition, both well studied in genetic analysis of tooth shape (Streelman et al. 2003; Streelman & Albertson 2006). Fertilized embryos were harvested from mouth brooding females and staged in days post-fertilization (dpf) according to Nile Tilapia developmental series (56). Embryos were raised to desired stages for ISH or chemical treatment and euthanized with buffered MS-222 for fixation in either 4% paraformaldehyde or 10% neutral buffered formalin.

### Clearing/staining and dentition staging series

Clearing and staining were performed according to previously published protocols (Fraser et al. 2013). Briefly, specimens were fixed in 4% paraformaldehyde for at least 48 h and rinsed with DEPC-H2O. The specimen were then protein digested in trypsin solution, and calcified tissue stained with Alizarin red S solution (1 g/50 mL potassium hydroxide [KOH]) for 45 min. Specimen were rinsed in water and placed in 2% KOH solution for a period of 24 h and graded through KOH/glycerin solutions to 100% glycerin with thymol. To stage the dentition, PC embryos from a single brood were sequentially sacrificed for analysis (Supplemental 1). Lower jaws were dissected, cleared and stained, and viewed under a Leica dissecting microscope from a dorsal vantage point. Teeth were analyzed upon eruption from 30 dpf until 120 dpf, as well as one parent PC fish and lower jaw teeth while alive under anesthesia (MS-222).

### Cichlid in situ hybridization

Primers for target probe sequence were designed using the published and annotated genomes of tilapia species *Oreochromis niloticus* and the published genome of Malawi cichlid (Loh et al. 2008; Brawand et al. 2014a). It has been reported that genomic sequence diversity across the Lake Malawi assemblage is 0.28%, less than reported values for laboratory strains of zebrafish (Loh et al. 2008), and riboprobes were reactive across Malawi cichlid species. Primers were created using the annotated cichlid genome, and genes were verified and named according to this assemblage (Brawand et al. 2014a). Target sequences were polymerase chain reaction amplified and cloned, and sequences have been published in previous works (Bloomquist et al. 2017, 2019). Riboprobes were synthesized and labeled with Digoxigenin (Roche) using the Promega System Sp6/T7. In situ hybridization was performed using previously published methods in whole-mount and visualized using a AP conjugated anti-digoxigenin antibody (Roche) to activate a NBT/BCIP (Roche) blue color reaction (Bloomquist et al. 2017). Specimen were embedded in chick albumin and cross-fixed with 2.5% glutaraldehyde followed by a post-fixed with 4% PFA. Histological sections were cut at 18–20 μm using a Leica Microsystems VT1000 vibratome and then mounted with glycerin for imaging using a Leica DM2500 compound microscope with 20–40 × objectives. All experiments were performed on four fish for replicates of three broods, providing a total of twelve animals for section and analysis for each gene in the study and for each species. The first position (closest to midline) on outer row teeth were examined, and gene expression patterns were consistent across replicates. Figures provided are representative of general patterns observed in these experiments.

### Small-molecule Notch inhibitor

Small molecule inhibition of the notch pathway was performed using previously published protocols on small molecule pathway inhibitors (Bloomquist et al. 2015). Here, a stock solution of DAPT ((2S)-N-[(3,5-Difluorophenyl)acetyl]-L-alanyl-2-phenyl]glycine 1,1-dimethylethyl ester, Tocris) was created at 10 μm by dilution in dimethyl sulfoxide (DMSO, MP Biomedicals) as a solvent. At 40 dpf, bicuspid MZ cichlid fish were kept in a 100-µm solution of DAPT in water for 24 h while in Erlenmeyer flasks at 28 °C on an oscillating platform culture incubator (Barnstead Max 4000). The fish were then removed from the solution, rinsed with water, and allowed recovery for 1 month before sacrifice and analysis.

## Results and discussion

To date, there have been limited studies on the progression of complex dental patterning in cichlids. Studies have demonstrated the early development of cichlid teeth and their resultant adult dentitions, but less is known about the transitional juvenile stage (Fraser et al. 2009, 2013). To best understand the formation of complex tooth shape in cichlids, a developmental series staging study was completed of *Petrotilapia chitimba* (PC), a species of Malawi cichlid chosen because of their complex adult dentitions with hundreds of long narrow inward curving tricuspid teeth in multiple rows (Supplemental 1).(Fryer & Iles 1972) This species was also chosen because of the robust brood size (some broods consisting of around 100 fish) that enabled progressive serial to sacrifice of a single brood. It is important to know that as the cichlid jaw grows, the very first teeth form at the most midline and labial positions (equivalent to central incisors in mammals) (Fraser et al. 2008). Teeth are subsequently added with growth of the fish both laterally to existing teeth, as well as in subsequent rows (Fraser et al. 2008). While tooth number and row number vary by species, this general pattern of adding teeth most lateral and in subsequent lingual rows holds true for most fishes. For example, in LF, adults typically possess around 500 teeth with four to five rows, MZ typically possess around 150–200 teeth with five to six rows, and PC may possess six to 10 rows with more hundreds of teeth. (Fraser et al. 2008, 2009; Bloomquist et al. 2015). Each generation of tooth can be tracked by monitoring the first position tooth, which is the largest midline outer row tooth. Here, brood mates were sacrificed in groups of 10 to analyze staging at different time points and the generation of tooth was easy to assess by determining the stage of shedding and eruption at this position. By 50 days post fertilization, the second to third generations of teeth were erupted and functionally present in the first (most labial) row of PC teeth, and the first generation of second row teeth had erupted (Supplemental 1). At this stage, the second row of teeth were simple and conical (first generation), while the outer row of teeth, which were in their second to third generation and had undergone one to two rounds of replacement and demonstrated some complexity in cusp morphology, particularly in the most midline teeth. As subsequent generations of teeth replaced their more rudimentary form, they became more complex in shape. By 60 dpf, the most midline teeth were in their third generation and a small lateral cusp was evident. By the fourth generation of teeth at 110 dpf, tricuspid teeth were present resembling the adult form of the PC dentition. In subsequent rounds of replacement, both lateral teeth and more posterior rows transitioned to the homogenous tricuspid form of the PC dentition (Supplemental 1).

### Signaling centers in cichlid tooth shape

**Fig. 1:**
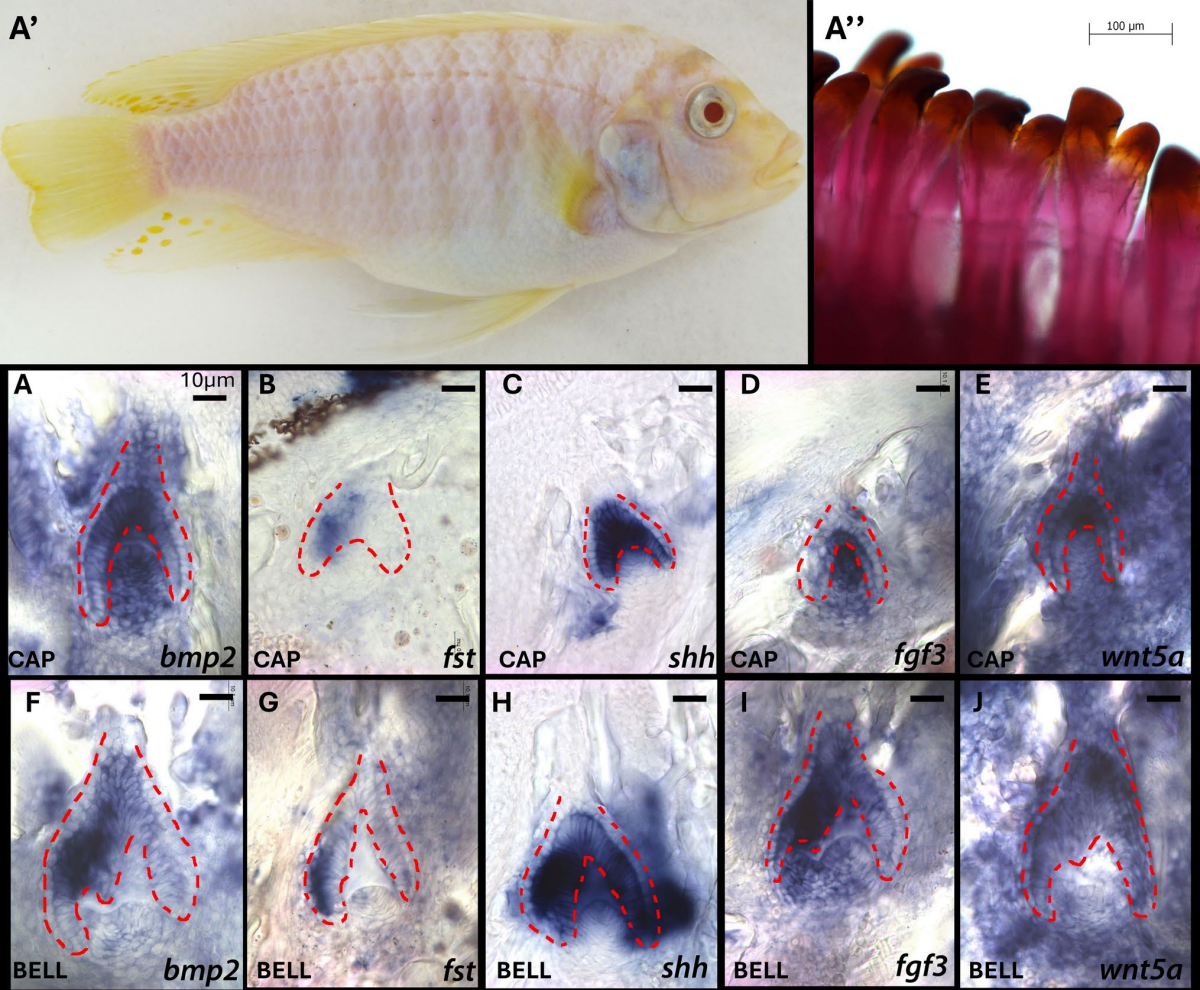
Picture of adult *Metriaclima zebra* (MZ) (A′) with phenotype of outer row bicuspid dentition that has gone through the clearing and staining process (A″). Expression of genes *bmp2*, *fst*, *shh*, *fgf3*, and *wnt5a* of bicuspid MZ teeth in the cap to bell stage of tooth development (top row) and the bell to secretion stage of tooth development (bottom row). These are vibratome sections in sagittal plane at 15 μm thickness, imaged at 20 × magnification. The oral cavity is oriented to the top of the image and lateral to the left of the image

**Fig. 2:**
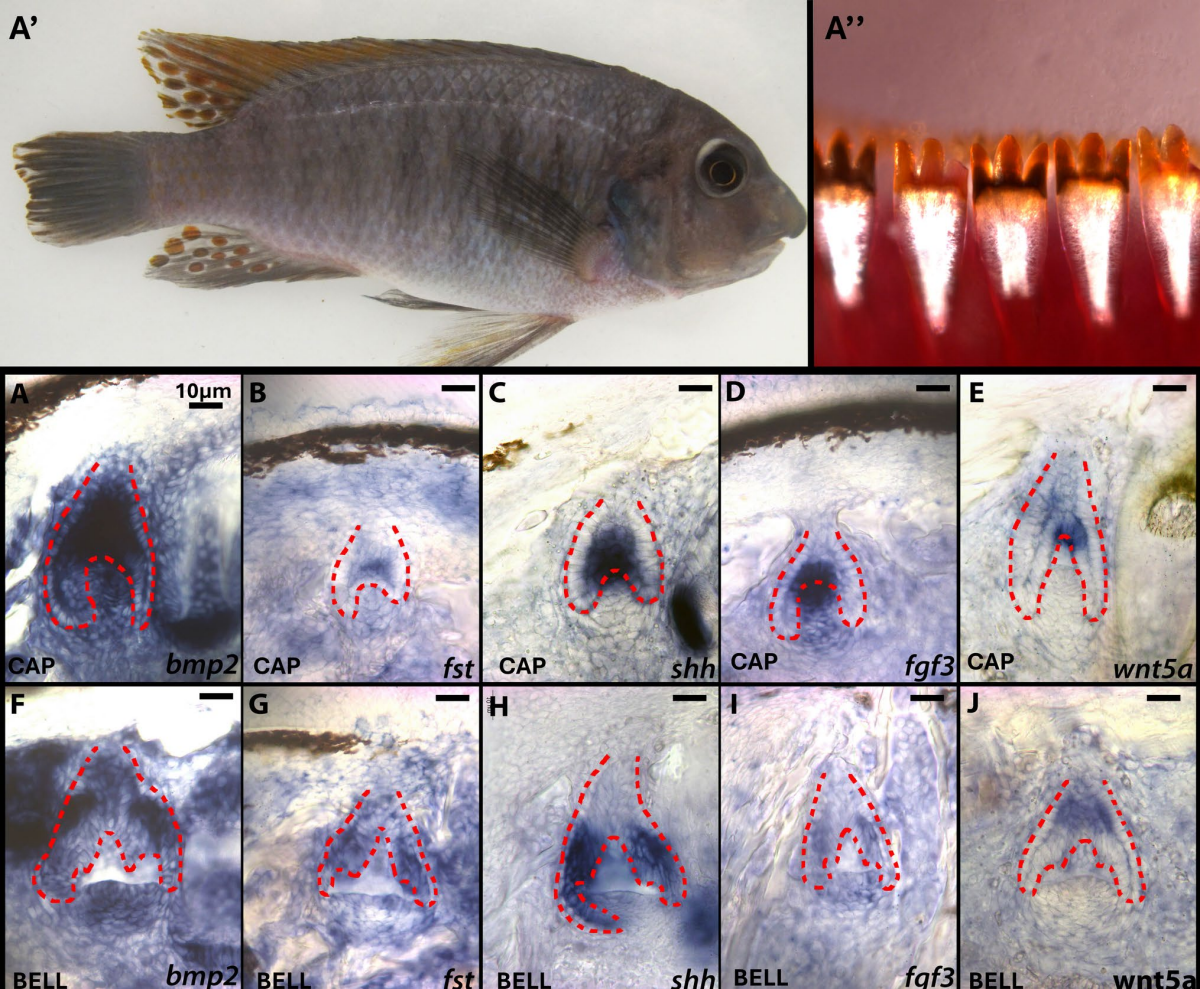
Picture of adult *Labeotropheus fuelleborni* (LF) (A′) with phenotype of outer row bicuspid dentition that has gone through the clearing and staining process (A″). Expression of genes *bmp2*, *fst*, *shh*, *fgf3*, and *wnt5a* of tricuspid LF teeth in the cap to bell stage of tooth development (top row) and the bell to secretion stage of tooth development (bottom row). These are vibratome sections in sagittal plane at 15 μm thickness, imaged at 20 × magnification. The oral cavity is oriented to the top of the image and lateral to the left of the image

**Fig. 3:**
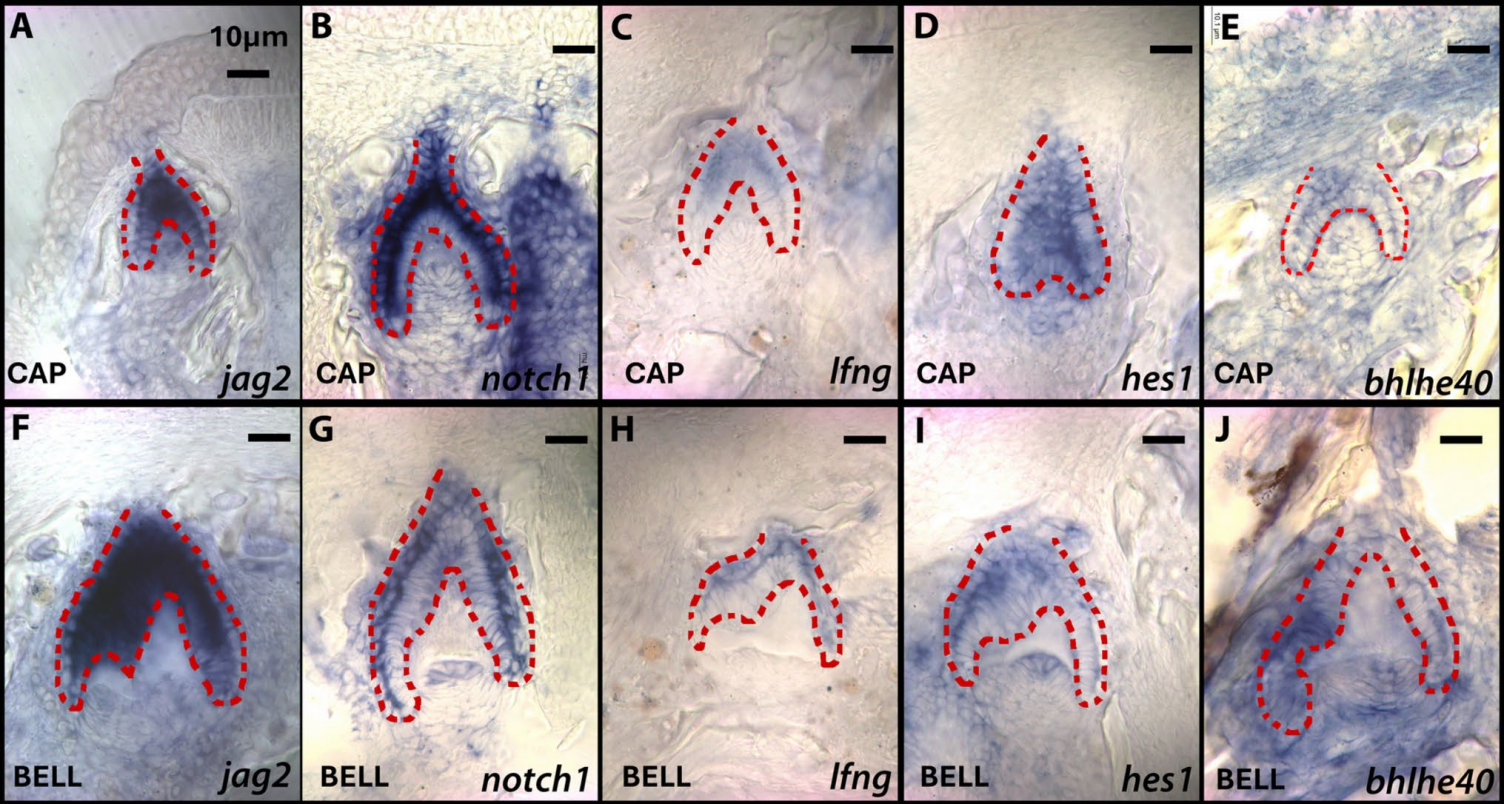
Expression of genes *jag2*, *notch1*, *lfng*, *hes1*, and *bhlhe40* of bicuspid MZ teeth in the cap to bell stage of tooth development (top row) and the bell to secretion stage of tooth development (bottom row). These are vibratome sections in sagittal plane at 15 μm thickness, imaged at 20 × magnification. The oral cavity is oriented to the top of the image and lateral to the left of the image

Developmental patterns of gene expression between *Metriaclima zebra* (MZ), a species with a bicuspid first row of teeth, and *Labeotropheus fuelleborni* (LF), a species with a uniform tricuspid dentition, were examined in order to compare gene expression patterns associated with shape determination of teeth. Using ISH, expression of *bmp2* at the cap to bell and bell to secretion stage was examined in both developing bicuspid (Fig. 1(A, F)) teeth of MZ and tricuspid teeth (Fig. 2(A, F)) of LF. Similar to that of mouse enamel knots, *bmp2* was expressed at a central focus of the presumptive primary cusp tip for both species (Figs. 1(A) and 3A), which, given the expression patterns as well as evidence of folding epithelium, can be considered an knot-like signaling centers. As the teeth matured, either a single asymmetrical second foci of expression was present, offset to the primary cusp, for bicuspid MZ (Fig. 1 (F)), or two symmetrical foci of expression were present flanking the primary cusp of tricuspid LF (Fig. 2(F)). The finding that the number of foci corresponds to the number of mature tooth cusps, while somewhat intuitive using what we know about the mouse, is significant as it suggests that fishes (and likely other non-mammalian vertebrates possessing complex tooth shape) employ knot-like signaling centers that likely contribute to tooth shape. Additionally, it is an example of natural variation in signaling center number corresponding to natural variation in cusp number between closely related species. Next, the Bmp family antagonist *fst* was examined. During the cap/bell stage of bicuspid tooth development, *fst* is expressed lateral and asymmetrically to the primary knot-like signaling center (Fig. 1(B)) and then further laterally and offset to the presumptive secondary knot-like signaling center as the tooth matures to the bell/secretion stage (Fig. 1(G)). In developing tricuspid teeth, the pattern is different, as *fst* is expressed in a sort of halo at the base of the primary knot-like signaling center (Fig. 1(B)) and later sandwiched in between the cusp tips (Fig. 2(G)). It is important to point out here that the inter-cusp signaling in bicuspid teeth is asymmetric and its offset precedes the appearance of the secondary knot-like signaling centers and furthermore is present beyond the initiation of the secondary knot-like signaling center. The persistence of the inter cusp signaling well into the secretion stages and past the initiation of the secondary cusp is interesting especially given that mature cichlid bicuspid teeth will always have a primary larger cusp located to the medial (dentally mesial, towards the midline) and a smaller secondary cusp located to the lateral location (dentally distal, away from the midline), even in the presence of hundreds of teeth. We next examined the Shh and Fgf pathways and found that analogous to primary and secondary enamel knots of the mouse, *shh* and *fgf3* ligands were expressed in primary knot-like signaling centers of bicuspid (Fig. 1(C, D)) and tricuspid (Fig. 2(C, D)) teeth and later in either a single offset secondary knot-like signaling center in bicuspid teeth (Fig. 1(H, I)) or two lateral and symmetrical secondary knot-like signaling centers in tricuspid teeth (Fig. 2(H, I)). In contrast to the knot-like signaling center markers *bmp2*, *shh*, and *fgf3*, the ligand *wnt5a* was expressed in the primary knot-like signaling center of both bicuspid (Fig. 1(E)) and tricuspid (Fig. 2(E)) teeth but was not present in secondary knot-like signaling centers and persisted in the bell/secretion stage. Both species of fish exhibit the same pattern of *wnt5a* gene expression and the knot-like signaling center persists, suggesting it may play a role in tooth morphology as in the mouse (Lin et al. 2011) perhaps as a primary cusp organizer, but it may not contribute to interspecific differences in cusp patterning.

### The Notch pathway and a novel candidate gene for dental diversity

During murine odontogenesis, the Notch pathway helps organize cusp number and integrate the BMP and FGF pathways through the Notch ligand *Jag2*, where explants treated with FGFs increased *Jag2* expression and those treated with BMPs decreased *Jag2* expression (Mitsiadis et al. 2005, 2010). In turn, *Jag2* heterozygous and homozygous nulls exhibit aberrant *Bmp4* expression, knot-like signaling center patterns, and severe dental abnormalities of shape (Mitsiadis et al. 2010)*.* In fact, members of the notch pathway play critical roles in proper tooth development throughout the process, including differentiation of the stratum intermedium lineage from dental epithelium (Harada et al. 2006) as well as the differentiation of odontoblasts and osteoblasts, and moreover in most dental developmental processes (Cai et al. 2011). Examples of genetic disease in notch pathway modulators resulting in aberrant tooth shape have been observed in humans, where mouse knockdowns of *Tspear* recapitulate tooth cusp and shape abnormalities seen in patients with frameshift or missense mutations of this notch pathway regulator (Peled et al. 2016). Basic Helix-Loop-Helix Family Member E40 (*bhlhee40*) is a member of the Hey/Hes related family Bhlh transcription factors downstream of Notch signaling (Fischer & Gessler 2007; Katoh & Katoh 2007). *Bhlhe40* is believed to primarily act by binding *Per1* in clock protein sites (Honma et al. 2002), which in mice which has also been described in the cap stage of mouse odontogenesis (Zheng et al. 2011). In uncontrolled cell regulation states such as in human thyroid cancer, *BHLHE40* can in turn influence and promote Notch signaling (Gallo et al. 2018), while in satellite cells of mice, *Bhlhe40* can actually antagonize Notch signaling (Sun et al. 2007), suggesting complex spatially and temporally specific reciprocal relationships exist between Notch signaling and regulation by *bhlhe40*. Employment of gene candidate *bhlhe40* to alter Notch pathway function may not be the only mechanism for evolutionary changes in cusp number between cichlid species but perhaps serve as a pathway modulator of tooth shape that permits ecologically driven morphological variation without severe associated pleiotropic effects.

**Fig. 4:**
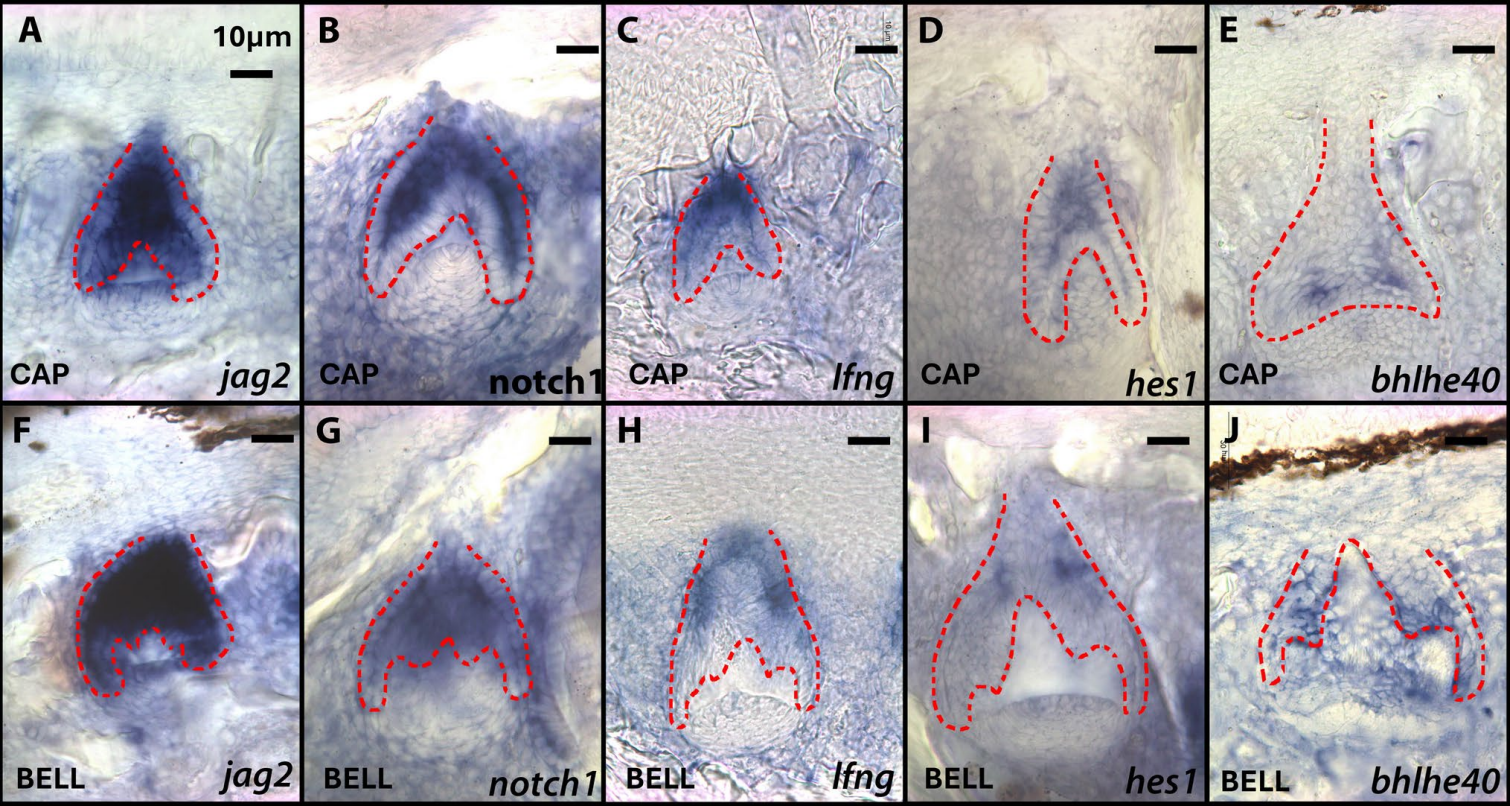
Expression of genes *jag2*, *notch1*, *lfng*, *hes1*, and *bhlhe40* of tricuspid LF teeth in the cap to bell stage of tooth development (top row) and the bell to secretion stage of tooth development (bottom row). These are vibratome sections in sagittal plane at 15 μm thickness, imaged at 20 × magnification. The oral cavity is oriented to the top of the image and lateral to the left of the image

Expression of Notch pathway ligand *jag2* was examined in developing MZ and LF teeth at cap/bell and bell/secretion stages of odontogenesis (Figs. 3A, F and 4A, F) and found to be similar between the two species. Here, *jag2* was expressed in the primary knot-like signaling center of MZ and LF and then transitioned into a more generalized pattern of expression across dental epithelium as the teeth matured, possibly due to the many roles that the Notch pathway and its different variations play across odontogenesis. While there seems to be a slight concentration of expression around primary knot-like signaling centers, receptor *notch1* and Notch inhibitor lunatic fringe (*lfng*) took on more of a general expression in between the inner and outer epithelium stratum intermedium of MZ and LF at both stages (Figs. 3 and 4B, C, G, H). Hes Family Bhlh Transcription Factor 1 *hes1* was first expressed in a generalized pattern between the inner and outer epithelium at cap stages (Figs. 3 and 4D) and then became localized to an inter cusp signaling foci simultaneously occupied by *bhlhe40* (Figs. 3 and 4I). In bicuspid MZ, *bhlhe40* was expressed lateral to the primary knot-like signaling center in the cap/bell stage on the side of the presumptive secondary cusp and maintained this proximity through the bell/secretion stage (Fig. 3E, J). However, in LF tricuspid teeth, two inter cusp signaling foci were expressed on either side of the primary knot-like signaling centers, well prior to the appearance of secondary knot-like signaling centers (Fig. 4E, J). This difference may suggest that together *hes1* and *bhlhe40*, possibly through Notch and cell cycling regulation, could be early organizers of cusp patterns and numbers and may contribute to cichlid dental polymorphisms.

### Notch pathway antagonism effect tooth shape determination in cichlids

**Fig. 5:**
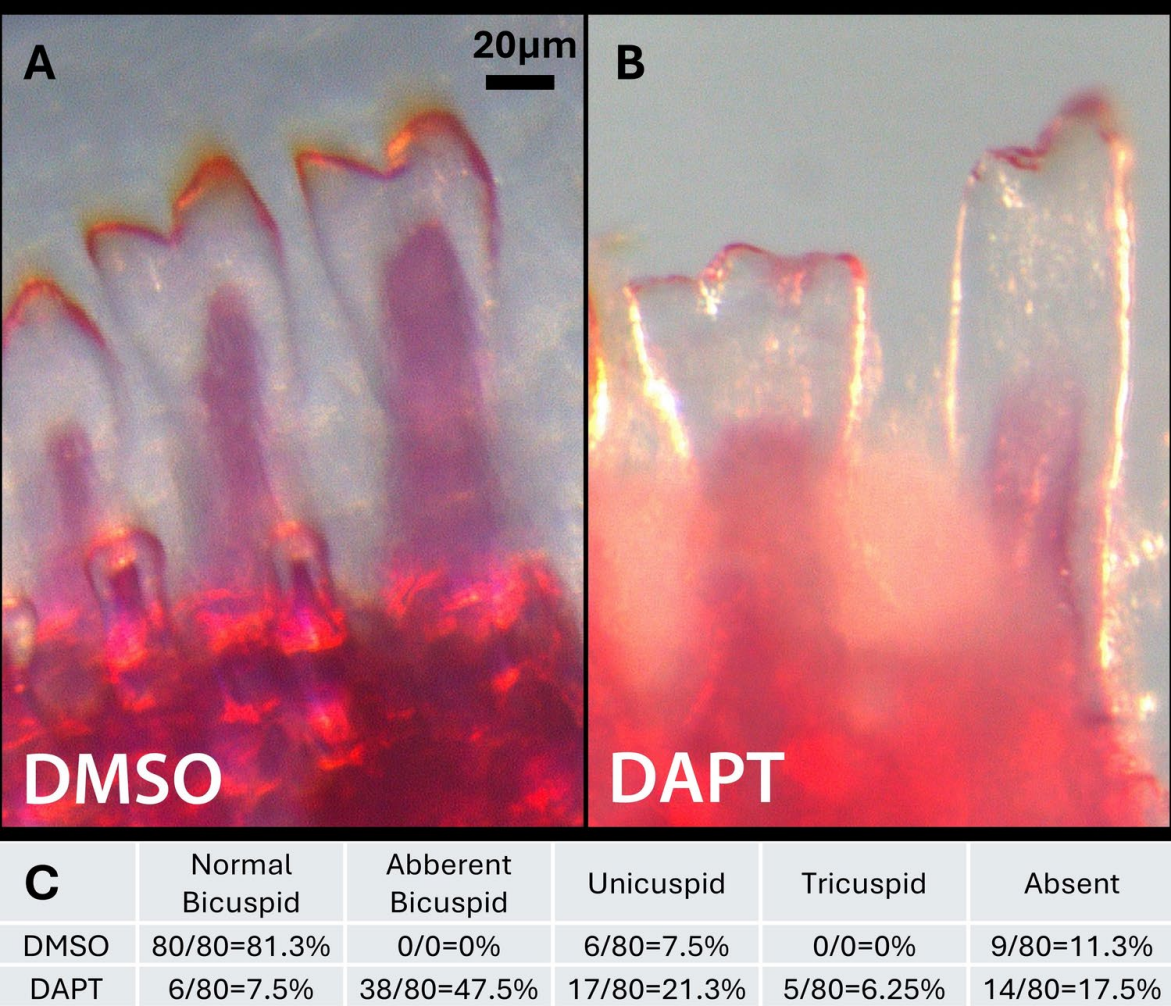
Image of cleared and stained lower left jaw midline outer row teeth in control MZ fish (bicuspid) to left of figure (**A**) and in DAPT treated teeth to right (**B**) showing shape perturbations, including transition from bicuspid to tricuspid tooth at second tooth position. A table of treatment data on phenotypes is presented (**C**) demonstrating basic statistics of dental patterning in first eight outer row teeth closest to the midline of lower jaw

While it is difficult to study functional analysis of specific genes directly in cichlids, the advantage of the natural “mutations” implied by interspecific variation is powerful. To test a possible role of morphogenetic regulation indirectly the Notch pathway was manipulated with small molecules. 40 dpf bicuspid cichlid fish (MZ) were bathed in small molecule Notch inhibitor DAPT for 24 h, and then permitted a recovery period of 1 month to allow developing teeth exposed to the molecule a period of recovery and eruption. Changes in cusp number, overall shape, and number of teeth were all noted, but the most interesting result was the conversion of an outer row position that should have been occupied by bicuspid tooth in MZ to an aberrant tricuspid tooth (Fig. 5). Of specimen in chemical manipulation experiment, all 10 (100%) displayed significant aberrations in first row tooth shape including unicuspid, malformed asymmetric bicuspid teeth, and tricuspid teeth, as well as missing teeth. The first four teeth from midline on either side of the outer row in the lower jaw were quantified (Fig. 5B). Six teeth out of 80 measured teeth demonstrated a tricuspid phenotype (3.75%) across three of the 10 treated specimen (30%) (Fig. 5). The pattern of cichlid tooth formation is predictable, and tooth shape is generated with such fidelity, that aberrations are rarely observed except in senescence and disease. In control experiments, no aberrant bicuspid or tricuspid teeth were noted (0%). Of note, there were several missing teeth (11.3%) attributable to replacement, wherein an earlier generation tooth had been exfoliated in preparation for the eruption of the next generation tooth. Similarly, several positions hosted unicuspid teeth (7.5%), as earlier generations of teeth tend to have more rudimentary unicuspid forms and later acquire more defined and complex tooth shape with each generation of replacement (Supplemental 1). Here, a single-day downregulation of Notch signaling was sufficient to create a phenocopy of a bicuspid to a tricuspid tooth in tooth positions at sensitive stages of development. No aberrations were noted in the control experiments, which were broodmates only exposed to DMSO. Hence, manipulation of the Notch pathway is adequate to generate polymorphism and alterations in genetic regulators may serve this purpose in nature.

## Conclusions

This study investigated the development of cichlid fishes that possess ecologically driven interspecifically diverse and intraspecifically constrained tooth shapes. Through a time-series and staging of broodmate cichlid jaws with hundreds of complex tricuspid teeth, PC add teeth in the midline and labial positions first and elaborate both posterior rows and additional teeth laterally with subsequent development (Supplemental 1). By 50 days post-fertilization, teeth in the most midline position and most labial rows in their second round of replacement demonstrate the beginnings of a tricuspid architecture with a more elaborate lateral cusp. By 78 days post-fertilization and after the third to fourth round of replacement, these positions are occupied by mature tricuspid teeth and by adulthood all teeth have transitioned to tricuspid teeth with more pronounced tricuspid morphology, ideal for scraping algae (Supplemental 1).

**Fig. 6:**
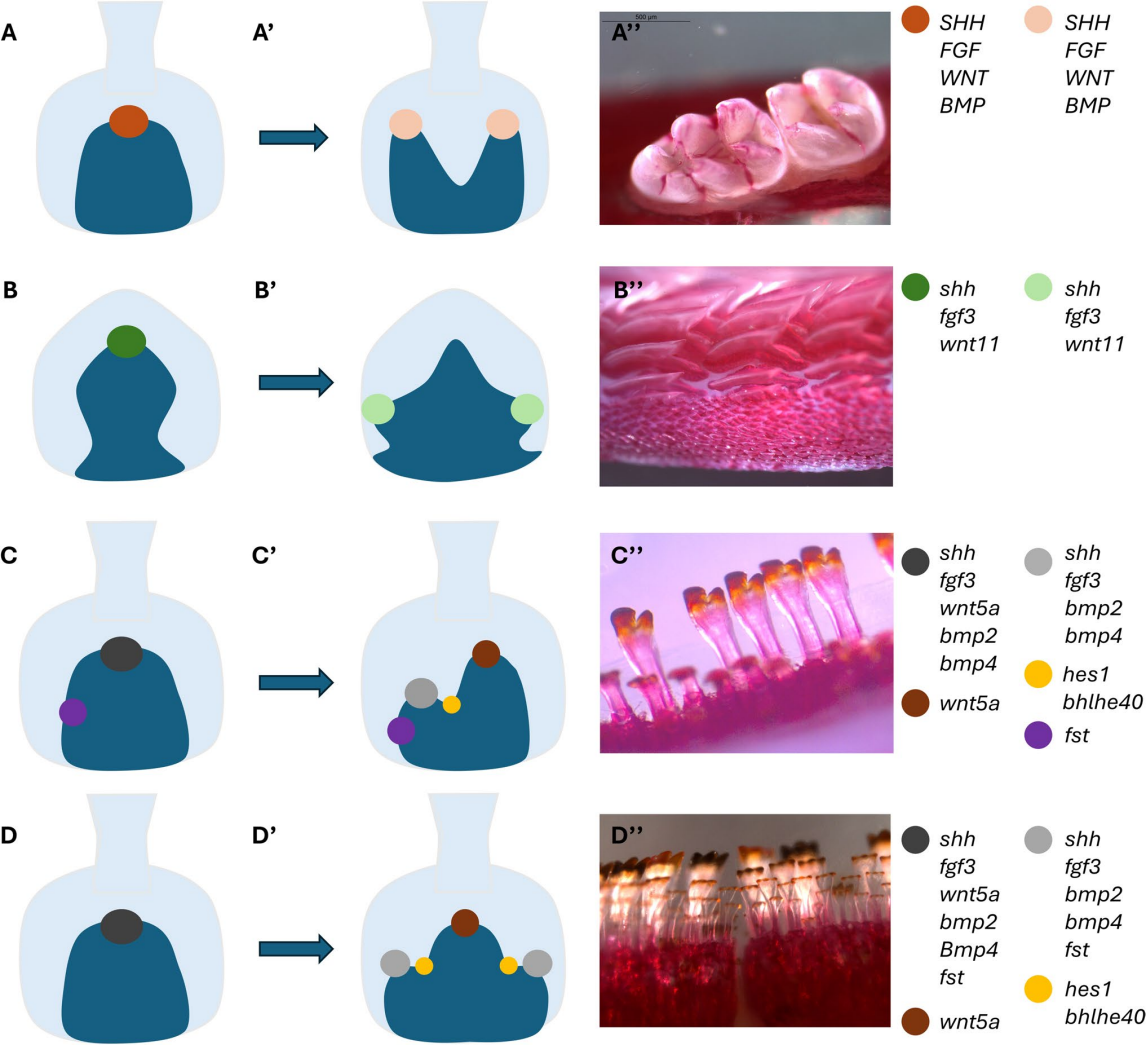
Cartoon illustration of knot and knot like signaling centers identified in mouse (**A**), shark (**B**), bicuspid MZ cichlids (**C**), and tricuspid LF (**D**). References provided in conclusion text. A, B, C, and D demonstrate primary signaling centers identified in respective species at cap stage. A′, B′, C’′, and D′ demonstrate secondary signaling centers identified in respective species at bell stage. A″, B″, C″, and D″ show mature and erupted phenotype and morphology of teeth for each respective species after previously described clearing and staining protocols. A color-coded legend is provided to left of schematic notating genes for each described signaling center

Next, informed by studies on tooth shape and enamel knots in mammals, the molecular underpinnings of complex tooth shape and dental diversity in cichlids were explored. By comparing gene expression patterns of developing teeth in cichlid fish with either bicuspid or tricuspid teeth, signaling centers at the tip of the developing central cusp were found, analogous to the mammalian primary enamel knot what can be termed a primary knot-like signaling center of the enameloid, as fishes do not possess enamel proper. In these centers, both folding of cells and a concentration of signaling including Bmp, Shh, Fgf, and Wnt factors share patterns with mammals that, to date, have not been well described in non-mammalian vertebrates. It has long been established that mouse primary and secondary enamel knots express Shh, WNTs including *Wnt5a*, FGFs, and BMPs including *Bmp2* (Fig. 6(A, A′)) (Jernvall et al. 1994, 1998; Jernvall & Thesleff 2000; Sunohara et al. 2022). Similarly, enamel knot-like signaling centers in ancestral shark species express *shh*, *fgf*, and *bmp2/4* at various stages of odontogenesis, and have been proposed to share shape determination similar to that of morphologically complex shark scales (Debiais-Thibaud et al. 2015a; Thiery et al. 2022).

Transient signaling centers at the tips of developing teeth in nonmammalian vertebrates have been identified in many species to date including geckos, alligators, zebrafish, and sharks (Handrigan & Richman 2011; Fraser et al. 2013; Tucker & Fraser 2014; Zahradnicek et al. 2014; Thiery et al. 2022). However, histologically, these signaling centers have not been described as knots owing to differences in cell behavior and most notably, a lack of identifiable cell migration and apoptosis in these animals that is a hallmark of enamel knot progression in mammals including the mouse (Matalova et al. 2005). Several theories exist for these differences including the notion that enamel knots are evolutionary derivations of these signaling centers, and perhaps tooth tip enamel knot like signaling centers in non-mammalian species such as sharks are ancestors of enamel knots, and moreover may predate teeth themselves as evolutionary remnants of patterning organs like scales (Thiery et al. 2022). Here, an alternative hypothesis is that some of these hallmarks such as apoptosis, may exist, but are less identifiable in the much smaller and lower cell volume signaling centers of non-mammalian teeth studied to date in fish and reptiles. Of note, apoptosis studies were attempted for this study but were not successful in the crowded bony jaws of cichlids. This is an area for further investigation and coupled with cell tracing studies may help prove or disprove the existence of true knots in cichlids and other fishes.

In this work, cichlids serve as models for natural polymorphisms in tooth shape and demonstrate differences of expression and patterning between developing bicuspid and tricuspid teeth of closely related species. Secondary signaling centers consistent with secondary knot-like signaling centers were seen with one in bicuspid species and two in tricuspid species, yielding two and three terminal cusps respectively (Fig. 6). Intriguingly, an early and obligatory lateral focus of Bmp inhibitor *fst* expression was reliably seen in bicuspid teeth both prior to and after the onset of secondary knot-like signaling center formation, but *fst* expression was concentrated within primary and secondary knot-like signaling centers in tricuspid teeth (Fig. 6). Another interesting note is that while other primary knot factors ceased to be expressed in primary signaling centers and became later expressed in secondary signaling centers in the bell stage, wnt5a persisted in the primary knot region but did not appear in secondary knots of either bicuspid or tricuspid fish (Fig. 6). The significance of this finding is undetermined but perhaps suggests that wnt5a plays a role in later cusp morphogenesis beyond cusp number determination. While factors of Notch expression were seen across the epithelium of both bicuspid and tricuspid teeth, the notch regulator *bhlhe40* was expressed early and in between presumptive cusps with one focus in bicuspid teeth and two in tricuspid teeth. Taken together these data imply that cichlid fish do indeed possess knot-like signaling centers of odontogenesis and early regulation of expression zone prior to secondary cusp formation may set the pattern for phenotypic dental diversity in shape and cusp number.

To test the role of the Notch pathway in shape determination, Notch signaling was antagonized during sensitive stages of tooth development with the small molecule inhibitor DAPT (Fig. 5). Surprisingly, a small but important number of outer row teeth developed an additional cusp, shifting from a bicuspid tooth to a tricuspid tooth in a species that otherwise does not possess tricuspid teeth. This phenotype is rarely, if ever observed in healthy developing fish with tricuspid teeth. Additional aberrations in shape presented as malformed bicuspid teeth and an increase in the number of both unicuspid and missing teeth as compared to controls (Fig. 5). Important in the interpretation of this data set is the fact that cichlid teeth are complex in the timing of the development, shape determination, and eruption of their jaws that each may possess hundreds of teeth each at staggard stages of development and replacement. In order to quantify the effects of chemical treatment, analysis was limited to the first eight teeth of the outer row of the lower jaw closest to the midline. However, the model is somewhat limited in the sense that it is almost impossible to know the timing of exposure of a given tooth position to small molecule inhibitors, as each developing replacement tooth will be in a different stage of development for a given animal. The increase in percentages of malformed teeth, unicuspid teeth, missing teeth, and most importantly the presence of tricuspid teeth in naturally bicuspid fish suggests that the Notch pathway is acting at multiple stages of replacement tooth odontogenesis, likely in both initiation stages as evidenced by missing teeth, as well as in shape determination. The presence of a small but important number of tricuspid teeth implies that fine tuning Notch signaling at very specific stages of shape determination is sufficient to generate polymorphism and may possibly underly species-specific dental patterning.

In summary, cichlid fishes with complex dental diversity exhibit surprising homology to mammals in tooth shape determination, and through manipulation of signaling centers, dental diversity can be generated in a laboratory setting to mimic species-specific patterning observed in nature.

## Supplementary Information

Below is the link to the electronic supplementary material.

Supplementary file1 (JPG 4483 KB)
