# Supplementary figures and images for: Developmental basis of natural tooth shape variation in cichlid fishes
Source: Naturwissenschaften. 2025 Jan 27;112(1):12. doi: 10.1007/s00114-025-01964-6 (PMC11772509; doi:10.1007/s00114-025-01964-6)

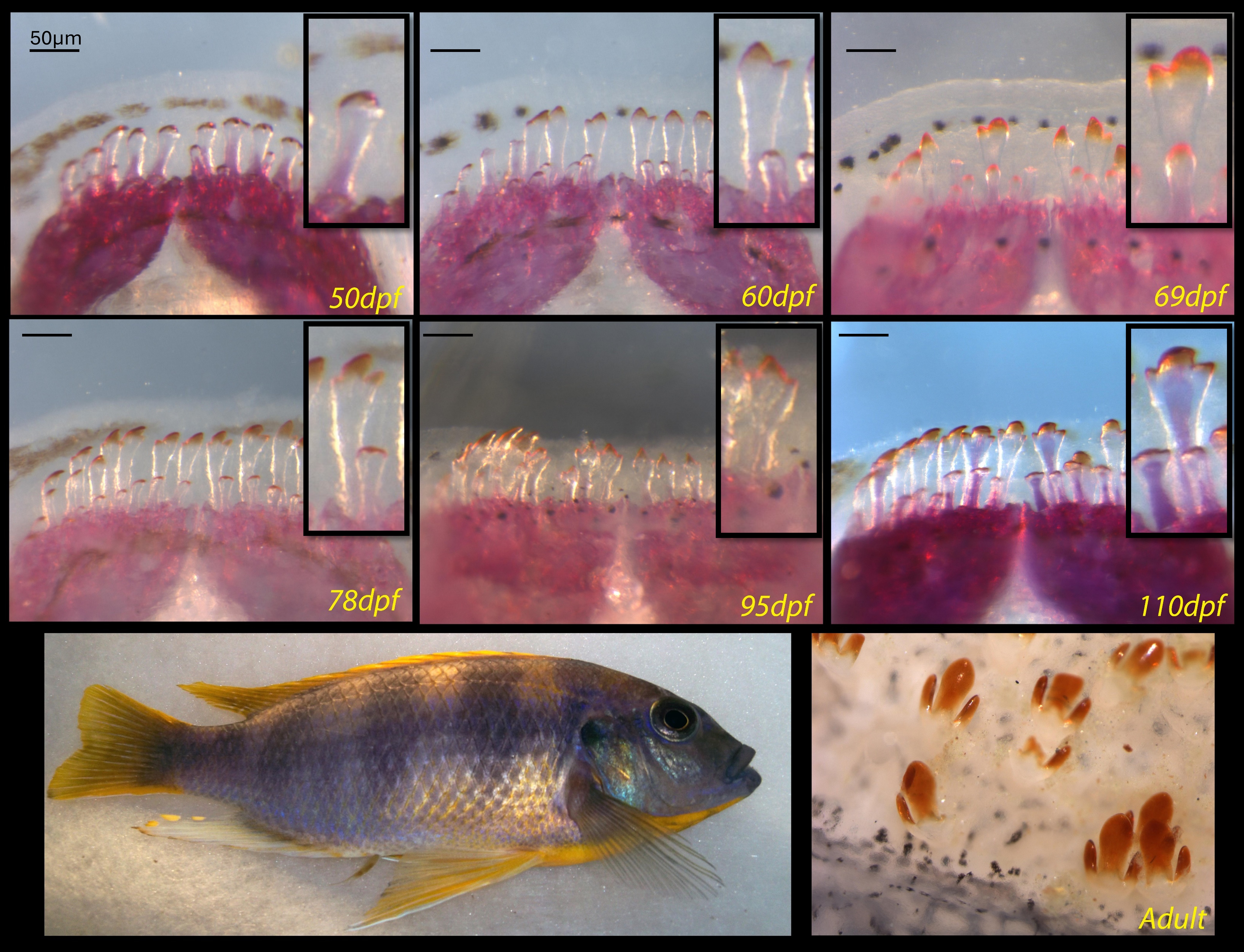

Supplement: Supplementary file 1 — Supplementary file1 (JPG 4483 KB) [file 114_2025_1964_MOESM1_ESM.jpg]
